# Response to Letter to the Editor from Sunaina and Mayilvaganan: Enhanced Recovery After Surgery (ERAS) Pathways for Aesthetic Breast Surgery: A Prospective Cohort Study on Patient-Reported Outcomes

**DOI:** 10.1007/s00266-025-04690-6

**Published:** 2025-02-04

**Authors:** Stéphane Stahl, Adelana Santos Stahl, Ana Cristina Seabra Robalo Gomes Jorge

**Affiliations:** 1 CenterPlast private practice, Bahnhofstraße 36, 66111 Saarbrücken, Germany; 2 Department of General-, Visceral-, Vascular-, and Pediatric Surgery, Saarland University Hospital, Kirrberger Straße, 66421 Homburg, Saar Germany

## Abstract

*Level of Evidence V* This journal requires that authors assign a level of evidence to each article. For a full description of these Evidence-Based Medicine ratings, please refer to the Table of Contents or the online Instructions to Authors www.springer.com/00266.

We appreciate your interest in our article, “Enhanced Recovery After Surgery (ERAS) Pathways for Aesthetic Breast Surgery: A Prospective Cohort Study on Patient-Reported Outcomes.”[1]

Below, we address the queries raised, which may be of interest to future readers:

**1. Did their cohort use any breast cancer patients since ERAS protocol for breast cancer patients shall be slightly different.**

**Answer:** None of the patients in our cohort had breast cancer. Our ERAS protocol was specifically designed for aesthetic breast surgery. The scope of our article did not extend to reconstructive breast surgery, for which there is already substantial evidence supporting the use of ERAS protocols.[2, 3]

**2. The number of patients do not add up to total in Table 2.**

**Answer:** As indicated in the legend of Table 2, the total number of patients with comorbidities and those taking medications does not sum to 48 (100%) because some patients suffer from more than one condition and, consequently, take more than one medication.

**3. The use of drains, especially in gynecomastia surgery (liposuction), is not mentioned.**

**Answer:** The systematic use of drains is not routinely recommended due to the lack of evidence. However, in cases where unusual diffuse bleeding was noted intraoperatively, two closed suction drains were placed as a precautionary measure. The drains were removed once the daily drainage volume consistently measured less than 30 mL.

**4. Were there any protocol deviations, such as antibiotic upgrades or prolonged intravenous fluid use?**

**Answer:** No, the surgical team strictly followed the ERAS protocol outlined in our study. Protocol modifications were not required, as the outcomes were excellent and patient satisfaction was high. For this reason, such updates were not discussed in the article.

**5. Isn’t revision surgery within 1 week itself a complication of ERAS protocol of performing surgery?**

**Answer:** The most frequent reasons for revision within one week may be hematoma or infection. Hematoma may be linked to insufficient hemostasis or coagulopathies, including pharmacologically induced or congenital disorders. Infection may be associated with smoking, diabetes, or intraoperative contamination. These complications are not specific to the ERAS protocol, which is designed to optimize recovery by emphasizing evidence-based practices such as prehabilitation, optimal pain control, early mobilization, and reduced perioperative stress. Complications directly related to the ERAS protocol may include inadequate pain control.
